# Correlation between TNM classification and malignancy histological feature of oral squamous cell carcinoma

**DOI:** 10.1016/S1808-8694(15)31308-2

**Published:** 2015-10-20

**Authors:** Antonio de L.L. Costa, Raimundo F. de Araújo Júnior, Carlos C.F. Ramos

**Affiliations:** ^1^ Ph.D., Professor, Program of Post- graduation on Health Sciences, Professor, Discipline of Oral Pathology, Department of Dental Sciences, UFRN; ^2^ Ph.D. studies under course, Program of Post-graduation on Health Sciences- UFRN/Professor, Disciplines of Histology, Physiology, Immunology and Pathology, Faculdade Santa Maria- PB; ^3^ Pathologist, responsible for the Laboratory of Clinical Pathology, Hospital Dr. Luiz Antônio, Natal, RN

**Keywords:** prognosis, oral squamous cell carcinoma, histologic grading, oral pathology

## Abstract

Histological staging of deep invasive margin of oral squamous cell carcinoma has a significant influence on survival of patients since the tumor cells are more poorly differentiated in this area and have high prognostic value.

**Aim:**

the purpose of the present study is to correlate TNM clinical classification with histopathologic characteristics (degree of keratinization, nuclear pleomorphism, invasion pattern and lymphoplasmocytic infiltrate) and histologic malignancy scores in 38 cases of oral epidermoid carcinoma in the lesion's deepest areas.

**Study Form:**

Retrospective clinical study.

**Material and Method:**

This is a retrospective study based on histological review of 38 cases of oral squamous cell carcinoma selected from the medical files of Hospital Dr. Luis Antonio, Natal - Rio Grande do Norte, Brazil. TNM clinical classification data were obtained from the analysis of the medical records. Two pathologists performed histological malignancy staging on routine 3 µm-thick sections of invasive tumor areas stained with hematoxylin and eosin. For statistical analysis, parametric (ANOVA) and non-parametric tests (Tukey; Pearson; Chi^2^) were employed.

**Results:**

We found significant correlation between TNM clinical staging and malignancy mean score (p = 0.001) and histopathologic parameters, such as nuclear pleomorphism (p = 0.016) and degree of keratinization (p = 0.025). Furthermore, there were also statistically significant correlations between lymphocytic infiltration (p = 0.016) and nuclear pleomorphism (p = 0.004) with TNM classification when grouped in two series: TNM I/II and III/IV.

**Conclusion:**

TNM classification, as well as malignancy mean score, had statistically significant correlation with degree of keratinization, nuclear pleomorphism and lymphocytic infiltration. These highly significant results indicated that histologically invasive areas may be primarily responsible for the clinical behavior of the tumor, and this may be important for the therapy of choice for oral squamous cell carcinoma.

## INTRODUCTION

In Brazil, incidence of malignant neoplasias of the oral cavity is substantially varied in different regions and this fact is probably due to local features concerning prevalence of risk factors^1^. Many risk factors are related to oral squamous cell carcinoma etiology, among which tobacco, betel, alcohol, virus and diet are included. These factors are associated with genetic inheritance and may have a carcinogenic effect on normal cells of respiratory and digestive systems^2^. Oral squamous cell carcinoma may occur anywhere in the mouth, although the most affected sites are tongue, lower lip and floor of the mouth. Features of these regions greatly facilitate carcinoma spreading to regional lymph nodes and/or distant organs^3^.

In patients with head and neck carcinoma, the prognosis is usually obtained based on TNM clinical classification, which is highly useful, specially to assess essential features of cancer, such as local extension, regional dissemination and distant metastasis^4^.

Patient's prognosis and survival^5^ may be determined with this system, which must include details of local anatomical characteristics to obtain data on grade of tumor involvement, as well as on distant metastases. However, the use of other prognostic markers is fundamental, once it helps to plan treatment and estimate patient's survival, which most of the times is reduced due to local recurrence or early lymph node metastases, added to existence of inaccurate prognostic factors^6^, ^7^. Histological staging of the deepest parts of oral squamous cell carcinoma directly influences patient's survival, once neoplastic cells in this site appear to be undifferentiated when compared to other tumor regions, constituting a valuable prognostic mean. Histological staging systems are very important, once they highlight the histopathological characteristics and the immunological relationship between tumor and host, predicting lesion's behavior in face of patient's response^8^, ^9^. Many studies were carried out to confirm correlation between clinical and histological parameters and prognosis. Keratinization grade, nuclear pleomorphism, number of mitoses, invasion mode and lymphatic infiltration were used by many authors as histopathological characteristics studied in superficial areas of tumor^11^. Byrne, in 1989, using the same histological markers (except for number of mitoses), suggested histological staging in invasive margins of lesion once he considered it to be the most representative region of neoplasia^11^.

Dib, Sabba (1994)^10^ have studied the medical records of 59 patients with oral squamous cell carcinoma between the period of 1960 and 1980. Histological and clinical variables such as sex, age, clinical staging (TNM) and presence of palpable lymph nodes were observed. These authors verified that clinical stage I patients presented higher overall survival rate, while those of clinical stage IV had lower survival. Moreover, tendency to keratinization and tumor thickness presented statistically significant correlation with overall survival^10^. On the other hand, other authors in similar studies - except for the idea that only the most invasive parts were to be graded - concluded that the invasion pattern was the histological staging system that had the most important correlation with clinical parameters, such as metastasis and recurrence^7^, ^11^.

Therefore, it was verified that this type of staging studies is more complex due to the heterogeneous features between oral squamous cell carcinoma subtypes and different behaviors this tumor has in different areas of oral mucosa. Furthermore, some clinical variables are not taken into account or investigated by the professional that performs the anamnesis of cancer patient. Considering that, this research study aimed at correlating TNM clinical classification with histopathological characteristics (keratinization grade, nuclear pleomorphism, invasion pattern and lymphoplasmocitary infiltrate) and the histological malignancy scores in 38 cases of oral squamous cell carcinoma in deep areas of the lesion, as described by Bryne (1998)^9^.

## MATERIAL AND METHODS

### Population

Consisted of all cases of oral squamous cell carcinoma of Hospital Dr. Luis Antonio in the city of Natal/RN, in the period between 1990 and 1995. After the project was evaluated and approved by the Committee of Ethics of Hospital Universitário Onofre Lopes, the medical records of all patients with oral squamous cell carcinoma were searched.

### Sample

The selected sample consisted of 38 patients with oral squamous cell carcinoma assisted and registered in the medical records of Hospital Dr. Luiz Antonio. Records' analysis included information on gender and age of patients, anatomical sites of lesions and clinical TNM classification. These data were registered in a pre-developed simplified clinical filing card. The cases were selected according to the following parameters:

• **Gender and skin color**: men and women, considering Caucasians, Mulattos and Black descendents.
• **Age**: in age ranges, starting from the 4^th^. decade
• **Site of lesion**: lesion was analyzed according to its localization in the side border of tongue, lower lip, mouth floor, oral pharynx or jugal mucosa.
• **TNM clinical staging**: the version established by the International Union Against Cancer was employed (Hermaneck et al.^12^, 1996; Neville et al.^13^, 1998): Stage I = T_1_ N_0_ M_0_; Stage II = T_2_N_0_M_0_; Stage III = T_3_N_0_M_0_, or T_1_, T_2_ or T_3_N_1_M_0_; Stage IV = any T_4_ lesion, any N_2_ or N_3_ lesion, or any M_1_ lesion.

Records with incomplete or confused data regarding clinical variables were disregarded and their respective paraffin blocks were not selected for further laboratory procedures.

Morphological analysis and histological malignancy stages of 3µm-thick tumor sections were conducted by two pathologists, using previously gauged optical microscopy and hematoxylin and eosin stain technique (H&E) in the deepest areas of the tumor. The sections were analyzed at the laboratory of Oral Pathology and further under light microscopy at the Department of Oral Pathology, UFRN.

Pre-selected cases of oral squamous cell carcinoma were assessed and classified by the histological malignancy staging system developed by Bryne (1998)^9^. The study consisted of evaluation of four morphological features: degree of keratinization, nuclear pleomorphism, invasion pattern and lymphocytary infiltrate. Total average scores of histological malignancy staging were obtained by summing the scores attributed to each morphological parameter divided by the number of parameters used, as to reduce occasional distortions at the final count. Therefore, following Bryne's system (1998)^9^, they considered 1.0-2.5 range as low malignancy score, and 2.6 to 4.0 range as high score (Table 1).

**Table 1:** Histological malignancy staging system according to Bryne (1998).

Morphological	Histological Score			
features	1	2	3	4
Keratinization degree	Highly keratinized (> 50% of cells)	Moderately keratinized (20-30% of cells)	Minimal keratinization (5-20% of cells)	No keratinization (0-5% of cells)
Nuclear Pleomorphism	Nuclear Pleomorphism (> 75% of mature cells)	Nuclear Pleomorphism moderately abundant (50-70% of mature cells)	Abundant nuclear pleomorphism 25% - 50% of mature cells)	Extreme nuclear pleomorphism (0-25% of mature cells)
Pattern of Invasion	Well-defined margins	Solid strings and/or inlets	Small cell groups n> 15.	Cellular diffuse dissociation and characterized in small and/or isolated cell groups
Lymphoplasmocytic infiltrate	High	Moderate	Discrete	Absent

### Statistical analysis

For results' analysis, following the Gaussian distribution, the parametric analysis was chosen (ANOVA). However, considering that some types are nominal qualitative variables, the non-parametrical Q-square, Tukey and Pearson tests were also applied. A 5% rejection index of null hypothesis was applied to all tests performed.

## RESULTS

**Figure 1:**
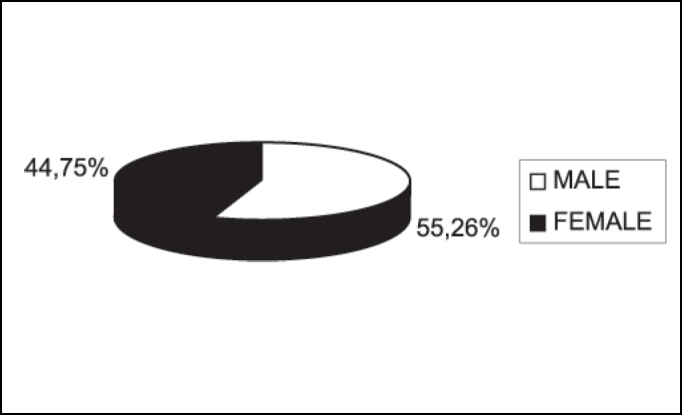
Number of patients distributed by gender.

**Figure 2:**
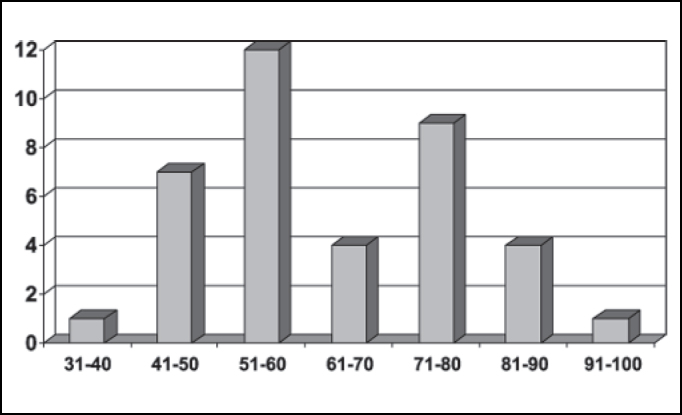
Number of patients distributed by age range.

Based on data analysis of 38 patients with oral squamous cell carcinoma presented in this study, the sample consisted of: 55.26% (21 cases) of men and 44.75% (17 cases) of women (Figure 1) between the ages of 40 and 93 years. The most prevalent age range was 51 to 60 years, represented by 57% of the cases (12 patients) (Figure 2).

**Figure 3:**
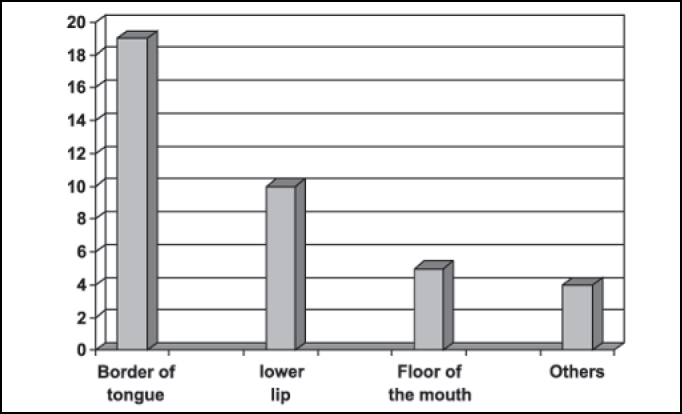
Number of patients distributed by anatomical site.

Regarding the anatomical site of the lesion, it was observed that the side border of tongue was the most affected region in 50% of the sample (19 cases), followed by the lower lip - 26.31% (10 cases), and floor of the mouth - 13.15%, (5 cases). Other areas such as the upper lip (2.63%), jugal mucosa (2.63%), oral pharynx (2.63%), and tongue center (2.63%) were also affected, summing 10.52% (4 cases) of the sample (Figure 3).

**Figure 4:**
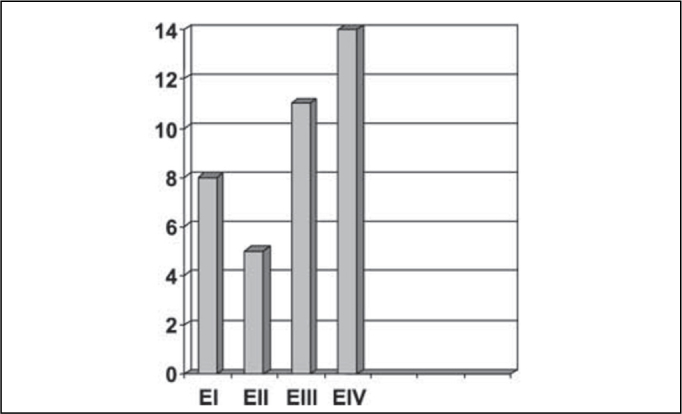
Number of patients distributed by TNM clinical stage.

Concerning TNM clinical staging, 14 patients were classified as stage IV, 11 as stage III, 5 as stage II and 8 as stage I (Figure 4).

As results using Tukey's statistical test were analyzed, a significant correlation was observed among combinations of TNM clinical stages when related to the following histopathological parameters: degree of keratinization (p = 0.05) and nuclear pleomorphism (p = 0.02). The same correlation was also verified with the average histological score (p = 0.01) (Table 2).

**Table 2:** Table 2. Descriptive statistics of variables, according to stage. Natal, RN. 2004

		n	Average	SD	CI (95%)		Min	Max
Variable	TNM				L.I.	L.S.		
Grade of	Stage I	8	1,75	0,89	1,01	2,49	1	3
Keratinization	Stage II	5	1,80	0,84	0,76	2,84	1	3
	Stage III	11	1,82	0,75	1,31	2,32	1	3
	Stage IV	14	2,57	0,51	2,27	2,87	2	3
	Total	38	2,08	0,78	1,82	2,34	1	3
Nuclear	Stage I	8	1,88	0,99	1,05	2,70	1	4
Pleomorphism	Stage II	5	2,00	0,00	2,00	2,00	2	2
	Stage III	11	2,36	0,50	2,02	2,70	2	3
	Stage IV	14	2,71	0,47	2,44	2,98	2	3
	Total	38	2,34	0,67	2,12	2,56	1	4
Invasion Pattern	Stage I	8	2,50	0,76	1,87	3,13	2	4
	Stage II	5	2,60	0,55	1,92	3,28	2	3
	Stage III	11	2,64	0,67	2,18	3,09	2	4
	Stage IV	14	3,14	0,66	2,76	3,53	2	4
	Total	38	2,79	0,70	2,56	3,02	2	4
Lymphoplasmocytic infiltrate	Stage I	8	1,88	0,64	1,34	2,41	1	3
	Stage II	5	1,80	0,84	0,76	2,84	1	3
	Stage III	11	2,00	0,89	1,40	2,60	1	3
	Stage IV	14	2,71	0,91	2,19	3,24	1	4
	Total	38	2,21	0,91	1,91	2,51	1	4
Mean Score	Stage I	8	1,99	0,67	1,43	2,55	1,5	3,5
	Stage II	5	2,10	0,58	1,39	2,81	1,5	2,8
	Stage III	11	2,14	0,49	1,81	2,47	1,4	2,7
	Stage IV	14	2,83	0,37	2,61	3,04	1,9	3,5
		Total	38	2,36	0,61	2,16	2,56	1,4

The statistical analysis of variance revealed that the degree of keratinization (p = 0.025), nuclear pleomorphism (p = 0.016) and average malignancy score (p = 0.001) had statistically significant correlation with TNM clinical staging (Table 3). Pearson's test showed correlation between the average histological scores and the isolated variables of TNM clinical staging (p < 0.005) (Table 4).

**Table 3:** Analysis of variance for independent variables in relation to stage. Natal, RN. 2004

Variable	Source of Variance	Square Sum	G.L.	Average squares	F	p
Grade of	Residual	5,398	3	1,799	3,523	0,025
Keratinization	Group	17,365	34	0,511		
	Total	22,763	37			
Nuclear	Residual	4,275	3	1,425	3,946	0,016
Pleomorphism	Group	12,278	34	0,361		
	Total	16,553	37			
Invasion	Residual	2,856	3	0,952	2,094	0,119
Pattern	Group	15,460	34	0,455		
	Total	18,316	37			
Lymphoplasmocytic	Residual	5,784	3	1,928	2,672	0,063
infiltrate	Group	24,532	34	0,722		
	Total	30,316	37			
Mean Score	Residual	4,985	3	1,662	6,470	0,001
	Group	8,731	34	0,257		
	Total	13,716	37

**Table 4:** Pearson's correlation test between markers and according to type of lesion studied. Natal, RN. 2004

Comparison	n	r	p
Mean score x Tumor size	38	0,443	0,005
Mean score x Node	38	0,488	0,002
Mean score x Staging	38	0,524	0,001
Tumor size x Node	38	0,191	0,251
Tumor size x Staging	38	0,875	< 0,001
Node x Staging	38	0,526	0,001

When TNM clinical stages were grouped into two series - I/II and III/IV - and related with the histopathological variables using the Q-square test, a correlation between these groups and the lymphoplasmocytic infiltrate (p = 0.016) and nuclear pleomorphism (p = 0.004) (Table 5) was also observed.

**Table 5:** Relation between presence of features and clinical staging of lesion. Significance obtained by Chi2 test. Natal, RN, 2004.

		Clinical Staging		Total	Qui^2^	p
		I and II	III and IV			
Grade of	High/Moderate	10 (76,9%)	15 (60,0%)	25 (65,8%)	1,088	0,297
Keratinization	Minimal/Absent	3 (23,1%)	10 (40,0%)	13 (34,2%)		
Invasion	Margins/Inlets	7 (53,8%)	7 (28,0%)	14 (36,8%)	2,455	0,117
Small group/Diffuse	6 (46,2%)	18 (72,0%)	24 (63,2%)			
Infiltrate	High/Moderate	11 (84,6%)	11 (44,0%)	22 (57,9%)	5,788	0,016
Discreet/Absent	2 (15,4%)	14 (56,0%)	16 (42,1%)			
Nuclear	None/Moderate	12 (92,3%)	11 (44,0%)	23 (60,5%)	8,354	0,004
Pleomorphism	Abundant/Extreme	1 (7,7%)	14 (56,0%)	15 (39,5%)		

## DISCUSSION

Prognosis of patients with oral squamous cell carcinoma is significantly varied due to different histological types and clinical features of this entity. Analysis of the TNM system and the histological staging has been adopted to plan treatment and provide better evaluation of patient's evolution. Patients with advanced tumors have poor prognosis, in which regional metastasis is the most important prognostic indicator^12^, ^13^.

Some clinical findings are discussed in this study to elucidate the relation between the events and the aspects approached, as well as to give rise to reflections that may contribute for new investigations.

Regarding clinical data, the sample showed that 55.26% of patients (21 cases) were men and that the cases within the 50-70-age range were the most affected ones (25 cases). No patients under 30 years old were registered, which is consistent with the literature available (Figures 1 and 2)^4^, ^10^, ^14^, ^15^, ^16^, ^17^.

Concerning anatomical, the side border of tongue (19 cases) followed by the lower lip (10 cases) were the most affected regions, which is in accordance with the data reported by other studies^4^, ^18^, ^19^ (Figure 3).

TNM clinical classification is one of the most used systems to predict the prognosis of oral squamous cell carcinoma. However, there are some divergences concerning tumor staging. It is a fact when dealing with primary tumors that advanced cases with infiltration of adjacent structures are hardly accessed at clinical examination^20^, ^21^. Regardless of the limitation of TNM clinical staging, its accuracy is around 70 to 80%. This percentage is higher when complementary exams, such as computed tomography and magnetic resonance, are used for classification and staging of neoplasias^20^. It is perfectly feasible to admit that results obtained by histological assessment are more accurate in prognostic settlement than essentially clinical exams, once microscopic findings provide information on events that are previous to those of clinical nature.

In order to assess an eventual correlation of TNM clinical staging with histological malignancy parameters and the average score proposed in our trial, a descriptive statistical analysis was conducted demonstrating statistically significant correlation between them^4^, ^9^, ^22^, ^23^, ^24^ (Table 2). However, these results changed when a variance analysis of independent histological parameters was performed in relation to the grade of TNM clinical staging, showing that invasion pattern (p = 0.119) was the only parameter that had no significant statistical relation with TNM clinical stage (Table 3).

The statistical analysis by Pearson's correlation test straightens the results of the statistics of variance, once there was correlation between the average histological scores and the clinical variables of TNM clinical staging (p < 0.005) when observed isolated^9^, ^22^ (Table 4).

The histological parameter “invasion pattern” is a valuable prognostic factor, once it reflects the capacity of cohesion of neoplastic cells^23^, suggesting that well-differentiated neoplasias invade within a well-defined margin pattern, while more anaplastic tumors infiltrate as small cell aggregates or isolated cells^19^. We understand that this explains its correlation with the prognosis.

Regarding TNM clinical stages of 38 cases distributed by I/II and III/IV groups and its relation with histological variables, a significant correlation was observed in these groups with lymphoplasmocytic infiltrate (p = 0.016) and nuclear pleomorphism (p = 0.004). These results may translate the possible effect of TNM clinical staging over tumor histological behavior, once III/IV patients presented poorer histological behavior. Contrary to our findings, Lopes et al. (2002)^15^ observed that the keratinization degree (p < 00.1) is the best histological parameter to determine the entity's clinical behavior and the risk of developing regional metastasis. In addition, the authors also verified that T_1_/T_2_N_+_ tumors presented high percentage of neoplastic cells with plentiful nuclear pleomorphism (p < 0.05), while T_3_/T_4_N_0_ tumors showed “invasion pattern” histological parameter in small-cell like groups in 65% of the cases (Table 5).

Histological staging of the most invasive margin, as suggested by Bryne (1998)^9^ and adopted in our trial, provides the best prognostic information on the neoplasm. Differently from former systems in which only the superficial part of the tumor was observed^25^, ^26^, ^27^, this author provides the pathologist^9^ with thorough information on anaplastic cells behavior.

## CLOSING REMARKS

By the end of our study, we concluded that keratinization degree, nuclear pleomorphism and lymphoplasmocytic infiltrate are strongly correlated to TNM clinical staging. The latter is strongly associated with malignancy scores, constituting important prognostic indicator. Therefore, there was statistically significant correlation between clinical staging (TNM), histological staging and malignancy scores.
